# Book Review: The Planetary Turn: Relationality and Geoaesthetics in the Twenty-First Century

**DOI:** 10.1177/1474474017732980

**Published:** 2017-10-02

In opening this sophisticated 12-chapter volume on the nascent discourse of planetarity in theory, literature and visual culture, editors Amy J. Elias and Christian Moraru explicitly acknowledge how their signal concept can be ‘frustratingly amorphous’ (p. xi). Yet, indeterminacy, it emerges, is planetarity’s provocative value. Building on Gayatri Spivak’s influential elaboration of the concept as a way to conceive world-scale entanglements, interactions and realms of difference without recourse to the kinds of conceptual reification perceived in globalisation theory, the book unites otherwise disparate formulations of planetarity through their common distance from totalising global overviews. While planetarity discourse shares globalisation’s disregard for demarcations of national culture, it cuts against the globalisation of capitalist calculability and fungibility.

Hence, *The Planetary Turn* is a valuable staging post towards alternative imaginations of geography: relational spaces of worldly reciprocity and flow that confound established coordinates. These geographical dimensions come out clearly in Hester Blum’s chapter which uses literary imaginations of oceans to present planetarity as a fluid apprehension of the world, exceeding projected standards of reference and measure and thereby sensitising readers to forms of otherworldliness in the worlds they cohabit. Planetarity theory’s recognition of alterity, counterposed to globalisation’s drive towards universal fungibility, is thematised in Robert Tally’s chapter (with the typically bracing title ‘Beyond the flaming walls of the world’), which affirms fantasy literature’s ability to project ‘otherworldly’ literary mappings of the planet. Other contributors, however, complicate Spivak’s go-to theory of planetarity as riven by alterity by accentuating modes of planetary commonality. In Iñárritu’s film *Babel*, for instance, Raoul Eshelman espies a ‘global relationality among humans that is unthinkable its entirety’ (p. 94), which Elias’ chapter imagines as a collective digital stewardship of the planetary commons. Along with Alan Kirby’s chapter on ‘cyber-placelessness’, Elias’ discussion also serves to ‘refute Spivak’s claims that planetarity should be set in opposition to computerisation’ (xxviii).

Perhaps the strongest part of this book is the editors’ introductory framing of planetarity, which offers a panoramic view of the concept’s intellectual history and its clashes and overlaps with theories of globalisation and cosmopolitanism. This is important reading for scholars of planetarity discourse, although Elias and Moraru’s periodisation of planetary culture as a post–Cold War phenomenon is perhaps too ruptural. (Interesting, then, are chapters treating earlier incarnations of the planetary, such as Wai Chee Dimock’s chapter on epic planetarity in adaptations of Gilgamesh or John Pizer’s chapter on planetary ramifications of Romantic translation theory, which trouble epochal periodisations in which planetarity suddenly succeeds statism). More pressing is the questionable extent to which some chapters engage planetarity; several pay lip service to the concept at their outset, but would otherwise fit nicely into volumes on cultural globalisation, eliding planetarity’s provocative deviance.

Still, in drawing together loose strands of planetarity discourse such that they coalesce around what Moraru’s chapter casts as an emergent ‘geomethodology’ of ‘reading for the planet’, *The Planetary Turn* should repay the interest of scholars working between geography and critical theory (pp. 211–244). Furthermore, Moraru calls us to ‘face the world’s face’ – that is, to recognise the alterity or fantastic otherworldliness of the planet beneath or beyond our synoptic reductions. Through this call, the book feeds into a larger poetic and political project of reimagining world geographies that exceed capitalist globalisation (p. 235).

**Simon Ferdinand** *Department of Literary and Cultural Analysis, University of Amsterdam, The Netherlands*

